# Supplementary material for: Origin, evolution and diversification of plant mechanosensitive channel of small conductance-like (MSL) proteins
Source: BMC Plant Biol. 2023 Oct 5;23:462. doi: 10.1186/s12870-023-04479-2 (PMC10552396; doi:10.1186/s12870-023-04479-2)

Supplementary Figure 1. Phylogenetic analysis of MSL proteins among fungi, protozoa, bacteria and plants.

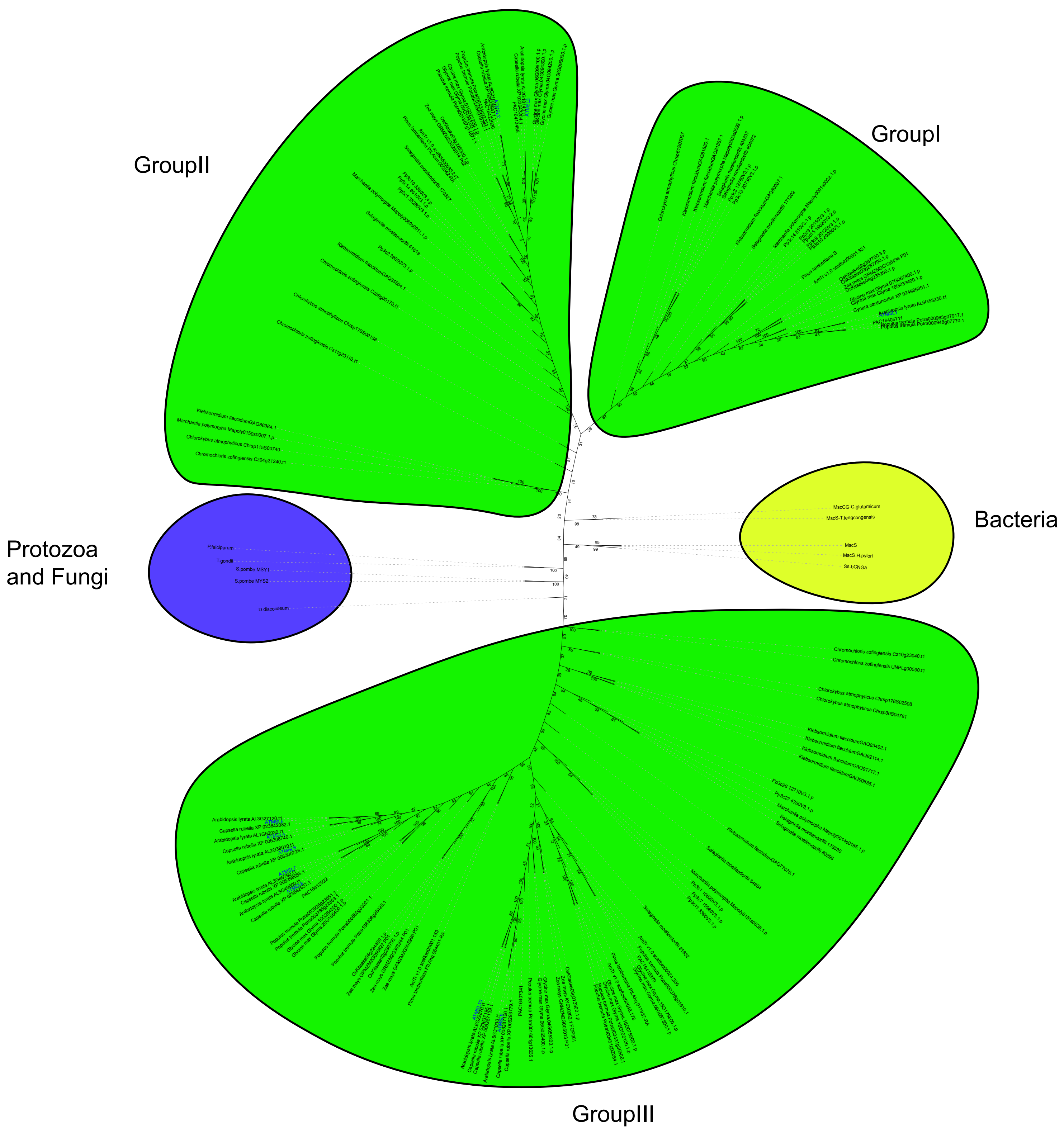

Supplement: Supplementary file 1 — Additional file 1: Supplementary Figure 1. Phylogenetic analysis of MSL proteins among fungi, protozoa, bacteria and plants. [file 12870_2023_4479_MOESM1_ESM.pdf]
