# Supplementary material for: Origin, evolution and diversification of plant mechanosensitive channel of small conductance-like (MSL) proteins
Source: BMC Plant Biol. 2023 Oct 5;23:462. doi: 10.1186/s12870-023-04479-2 (PMC10552396; doi:10.1186/s12870-023-04479-2)

Supplementary Figure 2. Phylogenetic relationship within the Group III of MSL proteins.

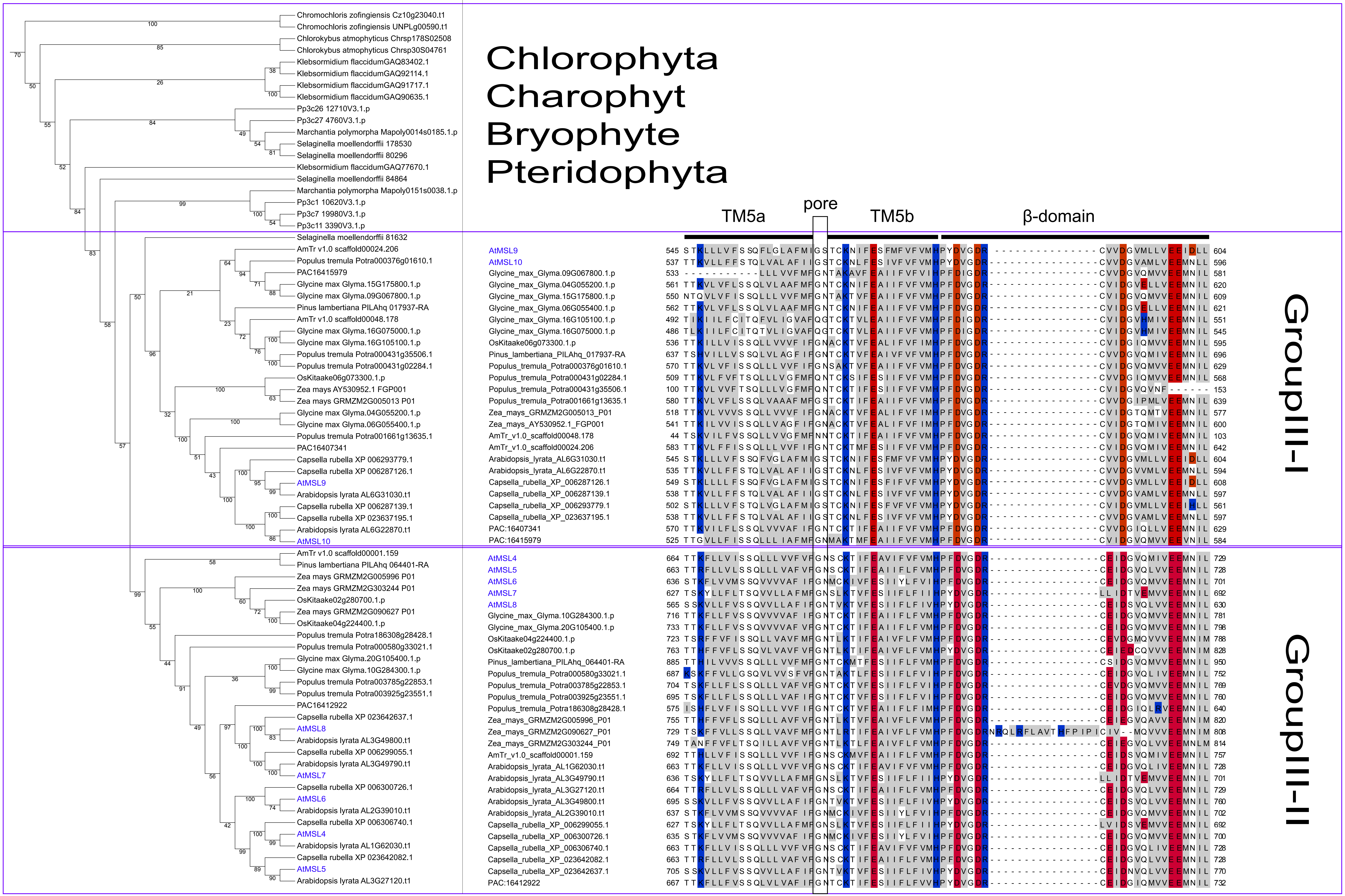

Supplement: Supplementary file 2 — Additional file 2: Supplementary Figure 2. Phylogenetic relationship within the Group III of MSL proteins. [file 12870_2023_4479_MOESM2_ESM.pdf]
