# Supplementary material for: Origin, evolution and diversification of plant mechanosensitive channel of small conductance-like (MSL) proteins
Source: BMC Plant Biol. 2023 Oct 5;23:462. doi: 10.1186/s12870-023-04479-2 (PMC10552396; doi:10.1186/s12870-023-04479-2)
Supplement: Supplementary file 4 — Additional file 4: Supplementary Table 2. The number of MSL proteins in the identified plants. [file 12870_2023_4479_MOESM4_ESM.docx]

**Supplementary Table 2. The number of MSL proteins in the identified plants.**

| Species name | Number of MSL |
| --- | --- |
| *Abies alba* | 2 |
| *Acer yangbiense* | 13 |
| *Actinidia chinensis* | 15 |
| *Alnus qlutinosa* | 11 |
| *Amaranthus hypochondriacus* | 15 |
| *Amborela trichopoda* | 4 |
| *Ammopiptanthus nanus* | 8 |
| *Ananas comosus* | 8 |
| *Anthoceros agrestis* | 6 |
| *Anthoceros punctatus* | 6 |
| *Antirrhinum majus* | 13 |
| *Apostasia shenzhenica* | 2 |
| *Aquilegia coerulea* | 8 |
| *Arabidopsis lyrata* | 10 |
| *Arabidopsis thaliana* | 10 |
| *Arabis alpina* | 6 |
| *Arachi ipaensis* | 9 |
| *Arachis hypoqaea* | 17 |
| *Artemisia annua* | 25 |
| *Asparagus setaceus* | 12 |
| *Asparaqus officinalis* | 5 |
| *Azola filiculoides* | 8 |
| *Begonia fuchsioides* | 14 |
| *Beta vulgaris* | 11 |
| *Boechera stricta* | 11 |
| *Bombax ceiba* | 17 |
| *Bonia amplexicaulis* | 10 |
| *Brachypodium distachyon* | 18 |
| *Brassica niqra* | 21 |
| *Brassica oleracea* | 22 |
| *Cajanus cajan* | 17 |
| *Calamus simplicifolius* | 13 |
| *Calohypnum plumiforme* | 11 |
| *Calotropis qiqantea* | 10 |
| *Camellia sinensis* | 22 |
| *Capsela rubella* | 12 |
| *Capsella grandiflora* | 11 |
| *Capsicum annuum* | 7 |
| *Capsicum baccatum* | 7 |
| *Cardamine hirsuta* | 10 |
| *Carex littledalei* | 10 |
| *Carica papaya* | 12 |
| *Carya cathayensis* | 7 |
| *Carya illinoinensis* | 9 |
| *Cenchrus americanus* | 5 |
| *Cercis canadensis* | 10 |
| *Chara braunii* | 2 |
| *Chenopodium quinoa* | 24 |
| *Chimonanthus salicifolius* | 8 |
| *Chlorokybus atmophyticus* | 5 |
| *Chromochloris zofinqiensis* | 5 |
| *Cinnamomum kanehirae* | 9 |
| *Citrullus lanatus* | 9 |
| *Citrus clementina* | 19 |
| *Citrus sinensis* | 20 |
| *Cocos nucifera* | 6 |
| *Coffea arabica* | 24 |
| *Coffea canephora* | 10 |
| *Corchorus capsularis* | 9 |
| *Coriandrum sativum* | 14 |
| *Cucumis sativus* | 10 |
| *Cucurbita maxima* | 12 |
| *Cucurbita moschata* | 11 |
| *Cuscuta australis* | 11 |
| *Cuscuta campestris* | 18 |
| *Cynara cardunculus* | 17 |
| *Daemonorops jenkinsiana* | 10 |
| *Daucus carota* | 18 |
| *Dendrobium catenatum* | 33 |
| *Dichanthelium oliqosanthes* | 6 |
| *Dimocarpus lonqan* | 12 |
| *Dryas drummondi* | 1 |
| *Echinochloa crus - gali* | 16 |
| *Elaeis quineensis* | 13 |
| *Eragrostis curvula* | 11 |
| *Eucalyptus grandis* | 26 |
| *Eutrema salsugineum* | 12 |
| *Fagopyrum esculentum* | 41 |
| *Fraqaria vesca* | 10 |
| *Fraxinus excelsior* | 16 |
| *Gelsemium sempervirens* | 9 |
| *Glycine max* | 16 |
| *Glycine soja* | 15 |
| *Gnetum montanum* | 13 |
| *Gossypium arboreum* | 17 |
| *Gossypium raimondii* | 15 |
| *Guadua angustifolia* | 4 |
| *Handroanthus impetiginosus* | 10 |
| *Helianthus annuus* | 20 |
| *Hordeum ulgare* | 10 |
| *Ipomoea trifida* | 9 |
| *Ipomoea triloba* | 9 |
| *Jatropha curcas* | 19 |
| *Kalanchoe fedtschenkoi* | 9 |
| *Klebsormidium nitens* | 10 |
| *Lablab purpureus* | 5 |
| *Lactuca sati a* | 16 |
| *Laqenaria siceraria* | 10 |
| *Linum usitatissimum* | 22 |
| *Liriodendron chinense* | 13 |
| *Macleaya cordata* | 10 |
| *Malus domestica* | 13 |
| *Manihot esculenta* | 12 |
| *Marchantia polymorpha* | 6 |
| *Medicago truncatula* | 13 |
| *Mesostigma viride* | 5 |
| *Mesotaenium endlicherianum* | 4 |
| *Mimosa pudica* | 14 |
| *Mimulus guttatus* | 12 |
| *Moringa oleifera* | 8 |
| *Morus notabili* | 17 |
| *Musa acuminata* | 8 |
| *Musa schizocarpa* | 7 |
| *Nelumbo nucifera* | 19 |
| *Nicotiana attenuata* | 10 |
| *Nicotiana tabacum* | 39 |
| *Nissolia schottii* | 9 |
| *Nymphaea colorata* | 5 |

| *Olea europaea* | 12 |
| --- | --- |
| *Olyra latifolia* | 6 |
| *Oropetium thomaeum* | 6 |
| *Oryza brachyantha* | 13 |
| *Oryza sativa* | 6 |
| *Panax ginseng* | 14 |
| *Panicum hallii* | 7 |
| *Panicum millaceum* | 14 |
| *Papaver somniferum* | 32 |
| *Parasponia andersonii* | 8 |
| *Penium margaritaceum* | 2 |
| *Petunia inflata* | 12 |
| *Phalaenopsis aphrodite* | 7 |
| *Phoenix dactylifera* | 19 |
| *Phyllostachys edulis* | 9 |
| *Physcomitrella patens* | 16 |
| *Picea abies* | 6 |
| *Pinus lambertiana* | 4 |
| *Populus tremula* | 13 |
| *Populus tremuloides* | 13 |
| *Populus trichocarpa* | 14 |
| *Primula eris* | 10 |
| *Prunus a ium* | 9 |
| *Prunus mume* | 11 |
| *Prunus persica* | 8 |
| *Pseudotsuga menziesii* | 5 |
| *Puccinellia tenuiflora* | 14 |
| *Punica granatum* | 16 |
| *Pyrus bretschneideri* | 22 |
| *Pyrus communis* | 13 |
| *Quercus suber* | 27 |
| *Raddia guianensis* | 6 |
| *Rhodiola crenulata* | 9 |
| *Rhododendron dela ayi* | 10 |
| *Rosa chinensis* | 13 |
| *Saccharum spontaneum* | 3 |
| *Selaginella moellendorffi* | 9 |
| *Setaria italica* | 6 |
| *Solanum lycopersicum* | 8 |
| *Solanum tuberosum* | 16 |
| *Sorghum bicolor* | 6 |
| *Spinacia oleracea* | 30 |
| *Spirodela polyrhiza* | 5 |
| *Taraxacum kok-saghyz* | 15 |
| *Tectona grandis* | 18 |
| *Theobroma cacao* | 13 |
| *Trema orientale* | 8 |
| *Trifolium pratense* | 13 |
| *Triticum aesti um* | 21 |
| *Vernicia fordii* | 15 |
| *Vigna unguiculata* | 10 |
| *Vitis vinifera* | 11 |
| *Volvox carteri* | 6 |
| *Xanthoceras sorbifolium* | 11 |
| *Zea mays* | 7 |
| *Ziziphus jujuba* | 17 |
| *Zostera marina* | 5 |
| *Zostera muelleri* | 7 |
